# imputeqc: an R package for assessing imputation quality of genotypes and optimizing imputation parameters

**DOI:** 10.1186/s12859-020-03589-0

**Published:** 2020-07-24

**Authors:** Gennady V. Khvorykh, Andrey V. Khrunin

**Affiliations:** Department of Molecular Bases of Human Genetics, Institute of Molecular Genetics of Russian Academy of Sciences, 2 Kurchatov sq., Moscow, 123182 Russia

**Keywords:** Imputation quality, Genotype, Haplotype, Cluster

## Abstract

**Background:**

The imputation of genotypes increases the power of genome-wide association studies. However, the imputation quality should be assessed in each particular case. Nevertheless, not all imputation softwares control the error of output, e.g., the last release of fastPHASE program (1.4.8) lacks such an option. In this particular software there is also an uncertainty in choosing the model parameters. fastPHASE is based on haplotype clusters, which size should be set a priori. The parameter influences the results of imputation and downstream analysis.

**Results:**

We present a software toolkit *imputeqc* to assess the imputation quality and/or to choose the model parameters for imputation. We demonstrate the efficacy of toolkit for evaluation of imputations made with both fastPHASE and BEAGLE software for HapMap and 1000 Genomes data. The discordance of genotypes received correlated well in both methods. Using *imputeqc*, we also shown how to choose the optimal number of haplotype clusters and expectation-maximization cycles for fastPHASE program. The found number of haplotype clusters of 25 was further applied for hapFLK testing that revealed signatures of selection at LCT region on chromosome 2. We also demonstrated how to decrease the computational time in the case of hapFLK testing from 3 days to 20 h.

**Conclusions:**

The toolkit is implemented as an R package *imputeqc* and command line scripts. The code is freely available at https://github.com/inzilico/imputeqcunder the MIT license.

## Background

Imputation is an in silico method that infers genotypes for undetermined or missed markers in study samples. It results both in harmonizing data sets and increasing the overall number of markers available for testing. The situation is common for genome wide association studies based on microarrays for genotyping portions of the genome variation. To increase the sample number and the power of the study, the datasets from different arrays are combined. Since they have different number of single-nucleotide polymorphism (SNPs), the joined dataset misses a lot of them. However, the missing genotypes can be replaced for their estimates provided with imputation.

The strategies of imputation are based on a heuristic, expectation-maximization (EM) algorithm or coalescent models [1]. They are implemented in the following softwares: fastPHASE [2], MaCH [3], IMPUTE2 [4], BEAGLE [5], and others. Imputation tackles two obstacles: time and accuracy. To balance the increase in computational burden necessary for large reference panels, two-step imputation procedure has been proposed. It includes an initial pre-phasing (i.e., haplotype estimation) of the studied genotypes with a subsequent imputation of reference panel markers into the estimated haplotypes.

The effect of imputation depends heavily on what is missing [6]. In each particular case one needs to control the quality of genotype reconstruction. A standard approach here is to apply masked data analysis, which involves three steps: genotype masking (making some known genotypes to be unknown), imputation, and comparison of the results obtained with the original ones. In general imputation touches either “hole filling” or reconstruction of entire SNP. Therefore two ways of masking is possible. We can either hide a proportion of genotypes or remove the whole SNP. Besides, the genetic markers can be masked randomly or intentionally.

Some softwares estimate the quality of replacement of missing genotypes. They have built-in options. However, the methods of assessing the quality are different. Thus, MaCH allows masking a certain portion of genotypes at random. Users apply the –mask option. The program estimates the allelic and genotypic error rates. The allelic rate is expected to be less than 5%. Authors of the program also advice to check the ratio of allelic to genotypic errors. It should be 2:1 since a small proportion of errors seems to be caused by imputation of one homozygote as the other one. It is also possible to estimate the imputation errors of MaCH by masking the whole SNPs, but extra scripts to generate the masked input files and count the errors after imputation will be required in this case. BEAGLE software provides *r*^2^ metric for each SNP and IMPUTE2 has INFO score, a metric for imputation quality for each SNP scaled from 0 to 1. Both reflects the correlation between imputed and true genotypes.

Other softwares, for example, last release of fastPHASE program (1.4.8), do not provide the users with built-in option to evaluate the quality of imputation. Additionally the users of this program meet a problem of choosing the optimal number of haplotype clusters (K). Scheet and Stephens called it a tricky statistical problem because Akaike and Bayesian information criteria did not work well [2]. They proposed to apply a masked data analysis to select K.

The number of haplotype clusters is also required for recently introduced hapFLK software [7]. It exploits haplotype cluster model for detection of signals of natural selection in genomes. The authors of hapFLK statistic emphasized that the distribution of hapFLK values can depend on the number of clusters thus pointing out the importance of choosing this parameter. At the same time, users of hapFLK cannot utilize information about the number of haplotype clusters from other software, for example, from BEAGLE, because the numbers identified varies along the genome [8]. Since such variation itself may be due to natural selection, hapFLK requires a fixed number of clusters for calculations.

The absence of ready-to-use solution for assessing the quality of genotype imputation in the case of fastPHASE pushed us to create a toolkit that would allow to estimate the error of imputation and select the optimal model parameters. Our research resulted in R package *imputeqc*. It utilizes the masked data analysis. The results of *imputeqc* testing demonstrated that it can be applied not only with fastPHASE but also with BEAGLE and other imputation softwares.

Below is the description of the package as well as its demonstration for datasets from HapMap [9] and 1000 Genomes Projects [10]. The quality of imputation made with fastPHASE was estimated and compared to that of performed with the most versatile tool BEAGLE. Both fastPHASE and BEAGLE are based on cluster models but of different implementation. The optimal numbers of haplotype clusters (K) and EM cycles were determined for fastPHASE. The K value was further applied for hapFLK test.

## Implementation

### The *imputeqc* project

*imputeqc* is an R package and accompanied scripts to estimate the quality of imputation of genotypes of diploid organisms. We approach the imputation quality as discordance between the imputed and true genotypes. The package supports two formats of input files: *.inp and Variant Call Format (VCF). The first one is applied in fastPHASE tool, while the second one is widely used for storing the DNA sequence variations.

*imputeqc* is based on masked data analysis that allows to estimate the imputation error. Comparing the errors over several calculations made with different inputs, the user can identify the imputation parameters statistically rather than heuristically. The examples of such parameters are the numbers of haplotype clusters and EM cycles.

The key functions of *imputeqc* are as follow:

1. **ReadfastPHASE()** parses fastPHASE *.inp files. There are two types of them: with ids of samples provided or not. The function loads both types. The alleles can be coded as letters or numbers, but the missing ones should be “?”. The function returns a character vector with strings representing the haplotypes. Two strings per individual.
2. **ReadVCF()** loads data from VCF files and returns a character vector with haplotypes. The function is built on top of *readVcf()* function from *VariantAnnotation* R package [11].
3. **GenerateMaskSet()** samples a set of masks, given a proportion of genotypes to be masked at each loci or the proportion of markers to be masked completely (*p*) and the number of masks (*n*). The masks are generated without replacement. This allows studying the space of possible variants of missing genotypes. Such an approach can be regarded as a general case of cross-validation method. The difference is in that *GenerateMaskSet()* allows to chose the number of data partitions (*n*), while the traditional cross-validation fixes this number. Before masking the function determines already existing missing genotypes. Thus, in the case of masking the genotypes, the overall missingness will be higher than *p*. The return value is a list of matrices with binary masks. The length of the list equals to *n*.
4. **ApplyMasks()** applies a set of masks to vector of haplotypes obtained with *ReadfastPHASE()* or *ReadVCF()* functions. The output is saved as *.inp or VCF files, correspondingly. The number of files created equals to the number of masks generated. The output files are ready for imputation with fastPHASE, BEAGLE or other appropriate tools.
5. **EstimateQuality()** counts the discordance between imputed and true genotypes as a proportion of genotypes imputed wrongly. By wrongly imputed genotypes we mean that either one or both alleles did not coincide with the original ones. Function returns a data frame for one set of test files with two columns: “discordance” and “id”. The first column contains the value of discordance. The second one has a user defined id. It is useful for a subsequent visualization. For example, there are several computations distinguishing by a model parameter. In this case, one can assign a value of model parameter to id argument of the function and build a graphic showing the descordance vs. model parameter. The second column is optional.

Besides R functions *imputeqc* project includes also some useful scripts:

- *make_test_files.R* automates creation of files with hidden genotypes or whole SNPs. It takes fastPHASE or VCF files, the desired number of different masks (*n*), the proportion of genotypes or SNPs to be hidden (*p*). The script outputs *n* files with artificially missed data.
- *hapmap2fastphase.R* converts HapMap data into fastPHASE format.
- *hapmap2plink.R* converts HapMap data into Plink format.

To access the scripts, install the *imputeqc* package. Then retrieve the path to the scripts by the command from R: system.file(“extdata”, package=“imputeqc”).

### Masked data analysis

Assume you have Plink files with genotypes and want to estimate the imputation quality as well as the optimal model parameters for fastPHASE program. The workflow proposed is as follow.

1. Convert Plink files to fastPHASE *.inp files, using Plink tool.
2. Generate a set of test files with *make_test_files.R* script which is enclosed to the package.
3. Impute the missing genotypes in each test file with fastPHASE.
4. Estimate the imputation quality with *EstimateQuality()* function in R.
5. Repeat steps 3 and 4 by varying one model parameter to be optimized.
6. Chose the parameter that minimizes the imputation error.

## Results

### Data preprocess

We used the same release of HapMap Project (Phase I, March 2005) as the authors of fastPHASE tool. Non-redundant genotypes were downloaded from ftp://ftp.ncbi.nlm.nih.gov/hapmap/genotypes/2005-03_16a_phaseI/full/non-redundant/. The data were converted into Plink format with a custom script *hapmap2plink.R*, which is also available within *imputeqc* toolkit. Having the file with pedigree information, we left 60 unrelated individuals from CEPH population. To be processed the genotype datasets should meet some thresholds (call rate, minor allele frequency, Hardy-Weinberg equilibrium). Using Plink v1.90b5.2 [12], we removed markers with MAF less than 0.01, not fitting Hardy-Weinberg equilibrium (*p*-value <10^−5^), and having genotype call rate less than 0.95. The duplicated by position markers were also removed. The number of SNPs thus left are given in Table 1.

**Table 1:** Dataset attributes and the discordances of genotypes imputated by fastPHASE and BEAGLE softwares

Population	Size	Chrom	SNPs	fastPHASE	BEAGLE
CEPH	60	7	28900	0.031 (0.004)	0.033 (0.003)
CEPH	60	22	13653	0.020 (0.001)	0.023 (0.001)
CEU, TSI, CHB, JPT	401	1	41524	0.0188 (0.0001)	0.020 (0.001)
CEU, TSI, CHB, JPT	401	22	7441	0.029 (0.001)	0.030 (0.001)

Genotypes of CEU, TSI, CHB, JPT and YRI populations were extracted with VCFtools 0.1.15 [13] from the archive Omni25_genotypes_2141_samples.b36.v2.vcf.gz downloaded from ftp://ftp.1000genomes.ebi.ac.uk/vol1/ftp/technical/working/. The list of SNPs containing 2384646 observations was filtered similar to HapMap data. Sex and mitochondrial chromosomes were removed. Only markers having “rs” in their id had been saved as Plink files. The number of SNPs and individuals filtered are given in Table 2.

**Table 2:** The number of SNPs and sample size for populations from 1000 Genomes Project after filtration

Population	Individuals	SNPs
CEU	102	613498
TSI	99	622338
CHB	100	581985
JPT	100	575606
YRI	101	632448

Five datasets were then merged by applying custom R scripts and Plink. The resulted file contained 512476 SNPs and 502 individuals. The Reynolds distances required for hapFLK calculations were further computed in R as follow:

$$\begin{array}{*{20}l} \Theta = \frac{\sum_{j=1}^{m}\sum_{i=1}^{a_{i}}\left(p_{ij} - q_{ij}\right)^{2}}{2 \sum_{j=1}^{m}\left(1 - \sum_{i=1}^{a_{i}}p_{ij}q_{ij}\right)} \end{array} $$

where *p*_*ij*_ and *q*_*ij*_ are the frequencies of *i-th* allele at *j-th* loci of two populations considered. They were estimated with Plink. For biallelic model *a*_*i*_=2. The obtained Reynolds distances are presented in Table 3. As it is seen from the table, the YRI population has the largest values of about 0.15 with CEU, TSI, CHB, JPT whereas these four populations are closer to each other as compared to YRI. Therefore, the YRI was chosen as outgroup for hapFLK testing. It was removed from the dataset, thus leaving 401 individuals for subsequent computations.

**Table 3:** The Reynolds distances for the populations from 1000 Genomes Project

	YRI	CEU	TSI	CHB	JPT
YRI	0.0	0.1515256	0.1473879	0.1745052	0.1758466
CEU	0.1515256	0.0	0.008708298	0.1095429	0.111366
TSI	0.1473879	0.008708298	0.0	0.1094925	0.1113009
CHB	0.1745052	0.1095429	0.1094925	0.0	0.01185164
JPT	0.1758466	0.111366	0.1113009	0.01185164	0.0

The kinship matrix was generated with *reynolds2kinship.R* script provided by hapFLK toolkit. The dataset was split into 22 files according to the number of autosomes. The chromosomes 1 and 22 were chosen for masked data analysis. The number of markers in the chromosomes are given in Table 1. To estimate the quality of imputation made with BEAGLE, we used the same files as for fastPHASE, while converting them into VCF format with Plink.

For each chromosome under consideration a set of 5 different test files containing artificially missing genotypes was generated with *make_test_files.R* script. It is a command line script built on *imputeqc* functions to automate the creation of files with missing genotypes. Ten percent of genotypes at each loci was randomly marked to be missing. The masks in a set do not repeat themselves, the script keeps a track of positions masked.

The VCF files containing 10% of missing genotypes were also generated with *make_test_files.R*, which recognizes automatically *.inp (fastPHASE format), *.vcf, and *.vcf.gz files.

### Demonstration

**Fig. 1:**
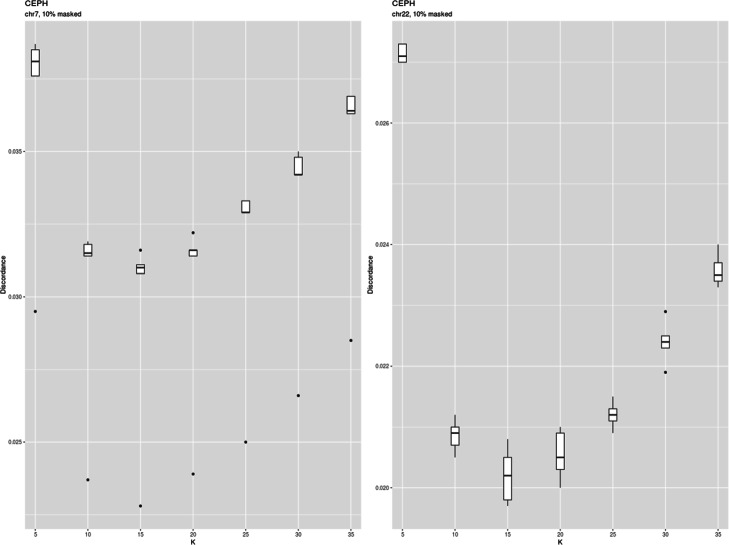
Discordance of CEPH genotypes from HapMap imputed with fastPHASE applying different number of haplotype clusters under 10% of missing genotypes

**Fig. 2:**
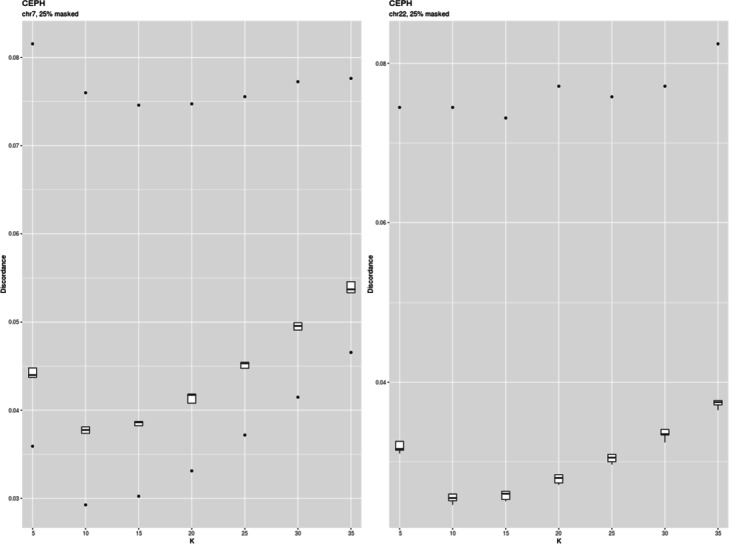
Discordance of CEPH genotypes from HapMap imputed with fastPHASE applying different number of haplotype clusters under 25% of missing genotypes

To test *imputeqc* efficacy we compared the results obtained by this tool with those published by the authors of fastPHASE. We ran the toolkit with fastPHASE (1.4.8) software on genotypes of 60 unrelated individuals from CEPH population of HapMap project by masking 10 and 25% of genotypes. In the case of 10% masked genotypes Paul Scheet and Matthew Stephens found the imputation errors to be 0.034 and 0.033 for chromosome 7 and 22, respectively. Using *imputeqc*, we obtained similar imputation error for chromosome 7 (0.031, SD 0.004) and lower value for chromosome 22 (0.020, SD 0.001) (Fig. 1). In the case of 25% masked data we also received discordances close to those of published by the authors of fastPHASE who reported 0.041 for chromosome 7 and 0.039 for chromosome 22 (Fig. 2).

Both chromosome 7 and 22 showed similar values of optimal K. But they differ depending on the missigness of genotypes. The discordance was minimal at K = 15 for 10% of masked data (Fig. 1) and at K = 10 for 25% of masked data (Fig. 2).

Since the haplotype cluster model is often applied to the pool of populations, we also demonstrated *imputeqc* for a dataset composed of genotypes from CEU, TSI, CHB and JPT populations of 1000 Genomes Project.

**Fig. 3:**
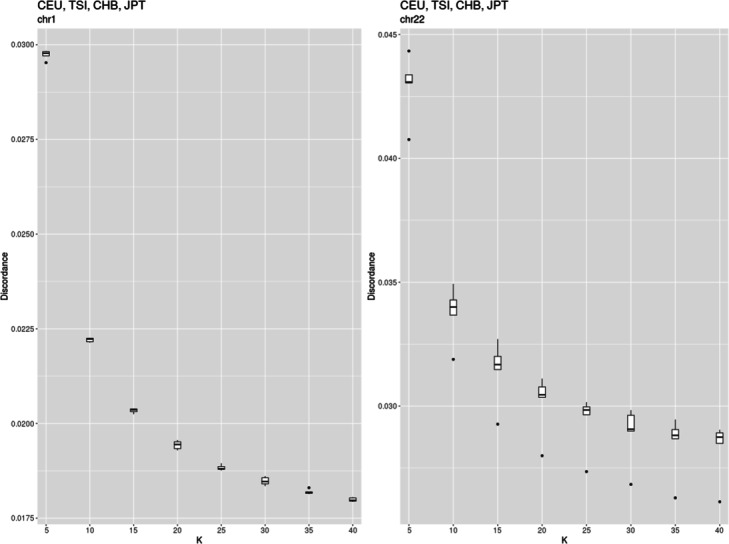
Discordance of pooled CEU, TSI, JPT, and CHB genotypes of chromosomes 1 and 22 imputed with fastPHASE applying different number of haplotype clusters

**Fig. 4:**
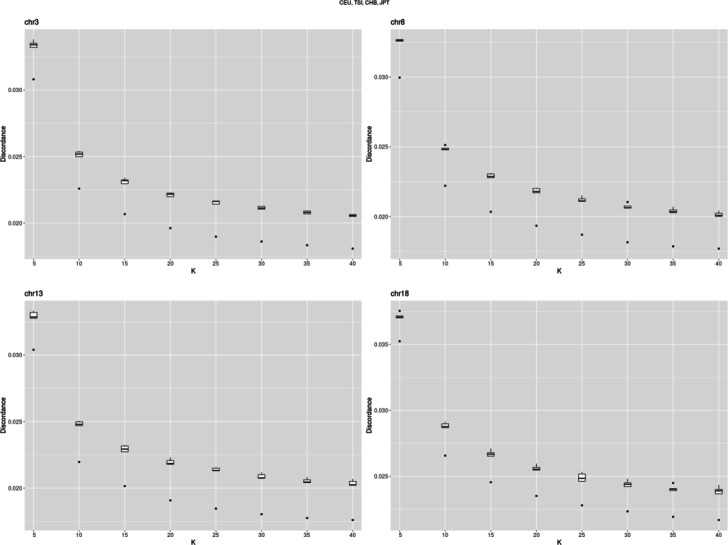
Discordance of pooled CEU, TSI, JPT, and CHB genotypes of chromosomes 3, 8, 13, and 18 imputed with fastPHASE applying different number of haplotype clusters

**Fig. 5:**
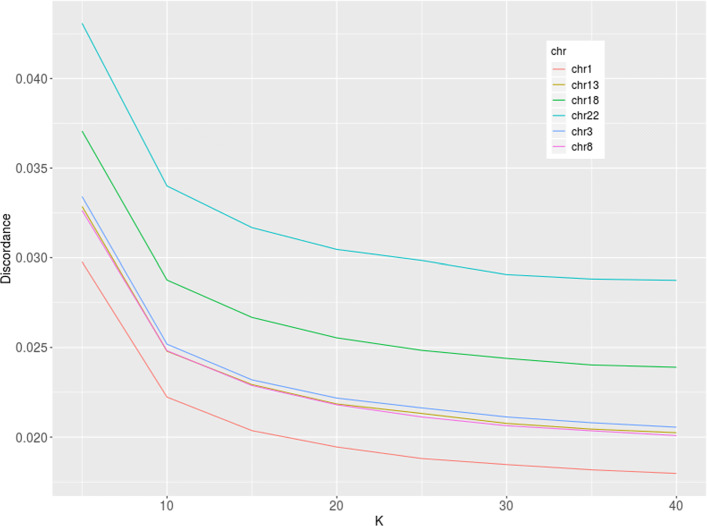
Medians of discordances of pooled CEU, TSI, JPT, and CHB genotypes of chromosomes 1, 3, 8, 13, 18, and 22 imputed with fastPHASE applying different number of haplotype clusters

**Fig. 6:**
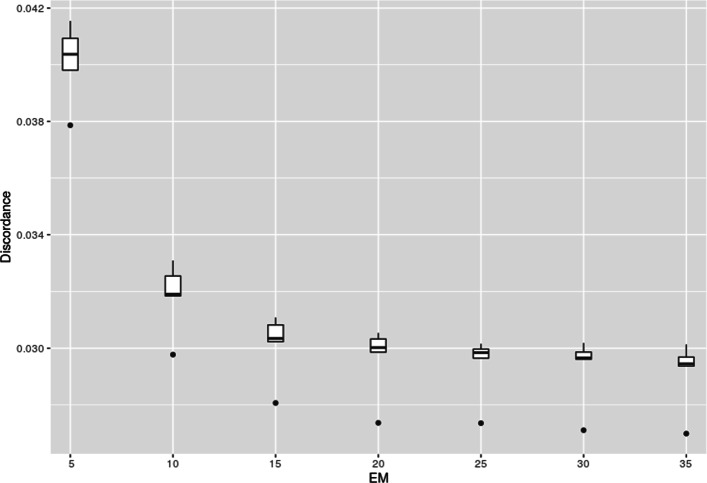
Discordance of pooled CEU, TSI, JPT, and CHB genotypes of chromosome 22 imputed with fastPHASE with different number of EM cycles

Firstly, we estimated the optimal numbers of haplotype clusters and EM cycles. Following the approach related to the “elbow” method (see Discussion), we found K to be 25 (Fig. 3), while the number of EM cycles to be 20 (Fig. 6). Then, applying these model parameters, we found the genotype discordance to be 0.0188 (SD 0.0001) and 0.029 (SD 0.001) for chromosome 1 and 22, respectively. Testing four other chromosomes (3, 8, 13 and 18) resulted in similar optimal K values. The discordances of genotypes were between those estimated for chromosome 1 and 22 (Figs. 4 and 5)

We also demonstrated the usage of *imputeqc* toolkit with BEAGLE (4.1) software. As fastPHASE it is based on cluster model, but the number of haplotypes clusters is adjusted dynamically. There is no need to optimize this parameter with extra calculations. BEAGLE showed similar to fastPHASE genotype discordance estimated with *imputeqc* for all datasets tested. The discordance is given as median followed by standard deviation in parentheses. Each value is calculated from five imputations of genotypes masked uniquely (Table 1).

**Fig. 7:**
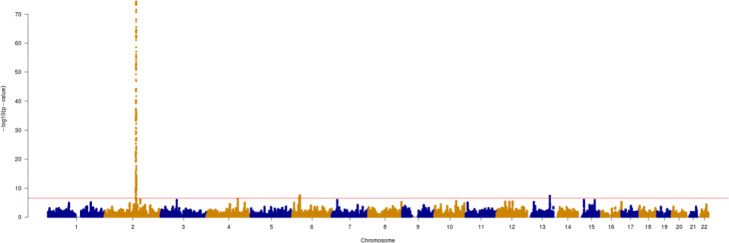
Manhattan plot of hapFLK *p*-values for the pool of CEU, TSI, CHB and JPT populations. Red horizontal line corresponds to 0.999-th percentile of *p*-values

**Fig. 8:**
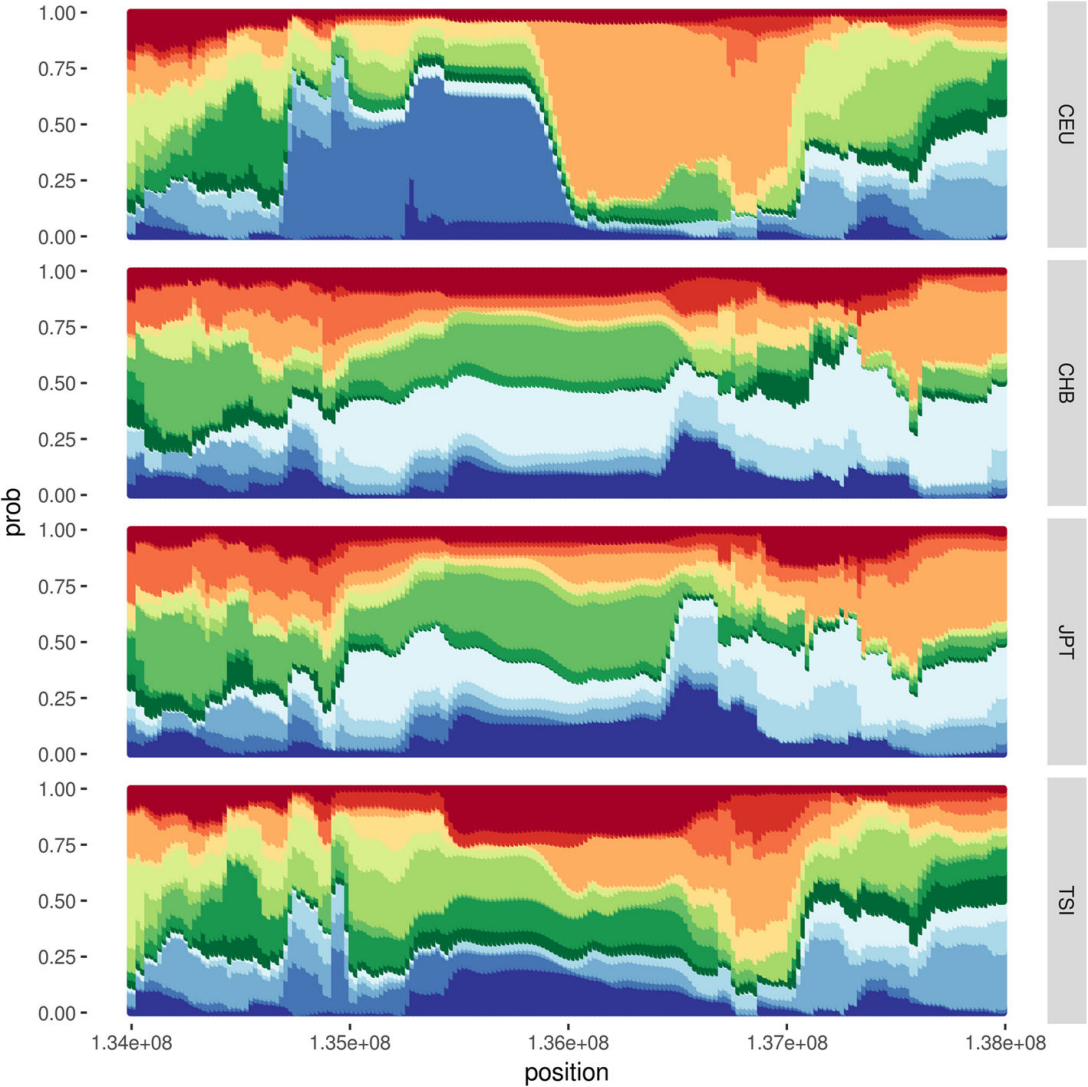
Haplotype cluster frequencies in 4 Mb region at chromosome 2 (LCT region)

The optimal number of haplotype cluster being known, hapFLK test for the search of footprints of selection can be performed. We ran it for a pool of CEU, TSI, CHB and JPT populations. YRI was chosen as outgroup. The *p*-values of hapFLK metric thus obtained are given in Fig. 7. They showed a strong signal at chromosome 2, position 137 Mb, which is known to be associated with a regulation of *LCT* gene. The analysis of haplotype cluster frequencies of 4Mb region around this signal indicated CEU population contributing essentially to the signal (Fig. 8).

### Imputation

Missing genotypes were imputed with fastPHASE 1.4.8 program applying the parameters as follow. The number of EM starts with different seeds was 10 in all calculations. The number of EM iterations was fixed to 20, while the K value varied from 5 to 40 with a step of 5. The K was fixed to 20, while the number of EM varied from 5 to 35 with a step of 5. Missing genotypes were also imputed with BEAGLE 4.1 program applying default parameters without reference genome.

### hapFLK calculations

hapFLK values were obtained with hapFLK (1.3.0) tool [7]. Ten model fit iterations, 25 haplotype clusters, and YRI population as outgroup were applied. *P*-values of hapFLK were estimated with *scaling_chi2_hapflk.py* available at hapFLK website [14]. The haplotype cluster frequencies were analysed and visualized with custom scripts in R. All plots in the paper were made with *ggplot2* R package [15].

### Computational time

We ran computations on Intel(R) Core(TM) i7-6800K CPU @ 3.40GHz machine with 64Gb RAM and 12 CPUs. Below are computation times in the case of a pool of CEU, TSI, CHB, and JPT populations. The creation of 5 masked files with *make_test_files.R* script took 5 minutes for chromosome 22 and 2 h for chromosome 1. The imputation with fastPHASE was quite time consuming. Thus, the imputation of 8 sets (K = 5, 10, 15, 20, 15, 30, 35, and 40) each having 5 masked files took 6 days for chromosome 22. In contrast, the imputation of 5 masked files with k = 35 took 6 days for chromosome 1.

We noticed that fastPHASE utilizes only one CPU. Therefore several runs of this program can be made in parallel. The GNU *parallel* [16] utility allows to do it. When it was applied, the imputation at K = 40 (5 masks) took 48 h for chromosome 1. The calculation of hapFLK metric required about 20 h.

## Discussion

An R package *imputeqc* is proposed and demonstrated for masked data analysis to estimate the quality of imputation and to select the optimal parameters for imputation and downstream analysis. Firstly, we wanted to be sure that the imputation error estimated by us was in agreement with literature data. For the purpose we reproduced the results published by the authors of fastPHASE tool. We masked 10 and 25% of genotypes. For both cases the estimated genotype imputation errors were similar to those obtained by Scheet and Stephens [2]. The same seemed to be also true regarding the optimum number of haplotype clusters. We estimated this parameter to be 15 and 10 for 10 and 25% of masked genotypes, respectively. However, the authors of fastPHASE made accent on its lower value (K = 8) that probably reflected their desire to keep the computational burden low.

Secondly, we demonstrated the application of *imputeqc* for the search of signatures of selection. It can be easily incorporated in a pipeline with hapFLK test. Here one needs to answer the question how many clusters should be considered in haplotype cluster model to perform hapFLK calculations in a particular pool of populations. That is why we merged datasets from 1000 Genomes Project (CEU, TSI, CHB, and JPT), generated several files with artificially missing genotypes, and imputed them with fastPHASE under different K values.

As seen from Fig. 5, the median of genotype imputation error decreases from 0.029 to 0.018, when K increases from 5 to 40 in the case of chromosome 1. Other chromosomes tested showed similar behavior but the discordance was higher. The shorter the chromosome was, the higher the discordance became.

We also found here that the error curve had an asymptotic shape at the range of K values studied, whereas in the case of one population it showed a clear minimum (Figs. 1 and 2). The change of the shape can be attributed to the bias-variance trade-off [17].

The higher the number of haplotype cluster, the more complex the model becomes. On the one hand, the raise in complexity drops the bias of the model. It tends to predict the outcome variable more accurately. On the other hand, the variance of predicted values becomes higher with increasing the error. The counter-play of two tendencies (bias and variance) results in a minimum of imputation error that we observed in the case of one population imputed (CEPH from HapMap).

When the object became more complex (e.g., four different populations were mixed), the minimum was not observed at the range of K values considered (Fig. 3). It is probably shifted to a higher number with K greater than 40. The positive influence of reduced bias predominates the imputation error observed. The error curve shows the permanent decrease. The question is how to choose the optimal K value in this case?

Determining the optimal number of clusters is a fundamental problem in Data Science. For example, the R package NbClust [18] provides 30 different indexes for estimating the best number of clusters. The authors of fastPHASE tested Akaike and Bayesian information criteria and found them to be inappropriate. To select K for fastPHASE, we propose an approach similar to “elbow” method [19]. The location of a bend in a plot of error against K indicates the optimal number of clusters. More clusters will not make the model better but will increase dramatically the computational time. Following this way, we found the optimal number of haplotype clusters to be 25.

To estimate the optimal K in the case of asymptotic behavior of the discordance curve, users can also apply more profound approach. For example, one can load the table with discordances created with EstimateQuality() function from *imputeqc* and process it with the function *elbow()* from GMD R package [20].

Package *imputeqc* has a practical benefit. There is a trade-off between accuracy and computational time, which depends on the model parameters. The computational complexity of fastPHASE algorithm is $\mathcal {O}\left (nMK^{2}\right)$, which means that it is liner dependable on the number of individuals and markers but square dependable on the number of clusters [2]. The optimization of K allows to reduce essentially the computational cost.

The toolkit proposed is designed in such a way as to optimize not only the number of haplotype clusters but also other parameters of the model. As illustration we considered the optimization of the number of EM steps. It should be enough for the likelihood to converge at its maximum value. The convergence can be estimated visually by plotting likelihoods or by assessing imputation quality with *imputeqc* at different values of EM steps.

The genotype imputation error demonstrated similar dependence on the number of EM cycles as that of haplotype cluster did (Fig. 6). Using “elbow” approach, we can conclude that the model converged in 20 iterations.

Thus, we demonstrated the usage of *imputeqc* not only for the estimation of genotype imputation error but also for the selection of optimal values of K and EM parameters to secure the computational time. Using the optimal values of K and EM steps, we further applied them for hapFLK testing of populations from 1000 Genomes Project.

The Manhattan plot of *p*-values estimated for hapFLK statistic showed a strong signal near *LCT* gene (Fig. 7). According to haplotype cluster frequencies, it is accounted by CEU population (Fig. 8). This observation is in a good agreement with the studies of haplotype diversity around *LCT* gene in human populations made with fastPHASE [21] and BEAGLE [22]. It should be also mentioned that hapFLK test has not yet been applied to the populations of humans. Our results showed its potential for the search of signatures of selection among humans.

Thirdly, *imputeqc* is flexible regarding the input files of different formats. Potentially it can be used to estimate the quality of genotypes inferred by any imputation program. To illustrate this, we estimated the quality of imputation made by BEAGLE software. We applied it to the same set of populations and found the errors to be close to those obtained with fastPHASE (Table 1).

Thus, *imputeqc* can be used for the estimation of imputation quality on real data sets, with the imputation softwares being different. It also provides a way to optimize the parameters of imputation models or test different inputs of the program applied. For example, the user can run imputation with different reference panels and compare the discordance of genotypes reconstructed.

To conclude, the *imputeqc* core functions are based on the standard R data classes [23]. Therefore, for deeper analysis of imputation quality it can be extended with functions available at other R packages. We validated our toolkit for fastPHASE and BEAGLE softwares but it is quite adaptive and can be used with other imputations tools allowing the users to assess the imputation quality independently by self-designing tests. However, in this case the scripts to make the input files may be required. We consider the toolkit as especially useful for the developers of new imputation algorithms as well as for those who study the factors influencing the quality of reconstruction of missing genotypes.

## Conclusions

A new open-source software toolkit, which includes an R package *imputeqc* and accompanied scripts, is proposed. It allows to estimate the quality of genotype imputation made with different tools as well as to determine the appropriate number of haplotype clusters for searching footprints of selection with hapFLK software. Other model parameters can be also optimized with *imputeqc*. The toolkit is freely available from GitHub repository by the link https://github.com/inzilico/imputeqc.

## Availability and requirements

**Project name:** imputeqc**Project home page:**https://github.com/inzilico/imputeqc**Operating system(s):** Platform independent**Programming language:** R**Other requirements:** R packages ggplot2, plyr, VariantAnnotation**License:** MIT License**Any restrictions to use by non-academics:** no restrictions

## Data Availability

*imputeqc* is freely available under the MIT license by the link https://github.com/inzilico/imputeqc.

## Abbreviations

EM: Expectation-maximization
MAF: Minor allele frequencies
SNP: Single-nucleotide polymorphism
VCF: Variant call format
